# Beliefs, attitudes and funding of assisted reproductive technology: Public perception of over 6,000 respondents from 6 European countries

**DOI:** 10.1371/journal.pone.0211150

**Published:** 2019-01-25

**Authors:** Bart C. J. M. Fauser, Jacky Boivin, Pedro N. Barri, Basil C. Tarlatzis, Lone Schmidt, Rachel Levy-Toledano

**Affiliations:** 1 University of Utrecht and University Medical Center Utrecht, Utrecht, The Netherlands; 2 Cardiff Fertility Studies Research Group School of Psychology, College of Biomedical and Life Sciences, Cardiff University, Cardiff, United Kingdom; 3 Department of Obstetrics, Gynecology, and Reproduction, Hospital Universitari Dexeus, Barcelona, Spain; 4 School of Medicine, Aristotle University of Thessaloniki, Thessaloniki, Greece; 5 Department of Public Health, University of Copenhagen, Copenhagen, Denmark; 6 Theramex HQ UK Limited, London, United Kingdom; China University of Science and Technology, CHINA

## Abstract

**Background:**

Fertility rates in Europe are among the lowest in the world, which may be attributed to both biological and lifestyle factors. Cost and reimbursement of fertility treatments vary across Europe, although its citizens enjoy wide access to fertility care. Since few regional studies evaluating public support for fertility treatment exist, we conducted the Listening IVF and Fertility in Europe (LIFE) survey to ascertain public perception of *in vitro* fertilization (IVF) and gamete donation as a treatment for infertility among European men and women.

**Methods and findings:**

This survey was distributed via an online questionnaire to 8,682 individuals who were voluntary participants in an online research panel residing in France, Germany, Italy, Spain, Sweden, or the UK. The survey covered items to determine respondents’ beliefs regarding IVF and its success, the need for public funding, the use of IVF among modern families with different lifestyles, and the support for gamete donation. Results were analyzed by age, country of origin, sex, and sexual orientation. A total of 6,110 (70% of total) men and women responded. Among all respondents, 10% had undergone IVF treatment and 48% had considered or would consider IVF in case of infertility. Respondents estimated IVF mean success rate to be 47% and over half of respondents believed that availability of IVF would encourage people to delay conception. Although 93% of respondents believed that IVF treatment should be publicly funded to some extent, a majority believed that secondary infertility or use of fertility treatments allowing to delay parenthood should be financed privately. Survey respondents believed that the mean number of stimulated IVF cycles funded publicly should be limited 2 to 3 (average 2.4). 79% of respondents were willing to pay for IVF if needed with a mean amount of 5,400 € for a child brought to life through IVF. According to respondents, mean minimum and maximum ages for IVF should be 29 and 42 years old, respectively. The current survey showed support for egg and sperm donation (78%), for IVF in single women (61%) and for same-sex female couples (64%). When analyzing the results per group (i.e., sex, age, sexual orientation, and countries), youngest age groups, homosexuals, bisexuals, German respondents, and men had similar overall positive attitudes and beliefs toward IVF and opinions on public funding. Perceived limits to availability were stronger in women.

**Conclusion:**

Overall, the survey results demonstrate a positive attitude among respondents in an online panel toward IVF, gamete donation, and support for public funding for fertility treatment. These findings could potentially drive discussions between patients and prescribers to explore IVF treatment and among legislators and payers to support public funding for these procedures.

## Introduction

Since the development of assisted reproductive technologies (ART), also referred to as medically assisted reproduction [1], couples with reduced fertility or advanced age, single women, and same-sex couples now have options to experience parenthood. The evolution of and increased access to fertility treatments have resulted in a substantial increase in number of babies born via *in vitro* fertilization (IVF) since the first IVF baby was born in 1978. According to the most recent available data published by the International Working Group for Registers on Assisted Reproduction and the International Committee for Monitoring Assisted Reproductive Technology, infants born annually via ART in countries reporting, increased approximately 40-fold from 11,323 [2] in 1989 to 404,364 in 2010 [3].

A wealth of information about ART exists on the internet [4], but current public perceptions of ART are still unknown because attitude surveys are outdated (almost >20 years ago), limited in size [5], or limited to those participating in gamete transfers [6–8], to other specific populations (such as infertile patients, students, childless individuals, or healthcare professionals), or to participants from individual countries [9–17].

The current survey, conducted online, involving a panel comprised of participants residing in France, Germany, Italy, Spain, Sweden, or the UK, queried over 6,000 men and women regarding IVF and gamete donation to ascertain their opinion concerning ART, its funding, and perceived limits towards its availability.

## Materials and methods

### Survey method

The web-based survey was conducted by Censuswide: The Survey Consultants (London, UK), whose methodology [18] complies with the Market Research Society (MRS) Code of Conduct rules (2010) [19]. The MRS rules are based on the International Chamber of Commerce/World Association of Opinion and Marketing Research Professionals (ICC/ESOMAR) International Code on Market, Opinion and Social Research Data Analytics [20]. Surveys were sent via an e-mail invitation to members of a panel originally recruited by sampling specialists. Panel members were general consumers presented with the opportunity to voluntarily join Censuswide panels using opt-in plus validation processes. Panel members also completed a profiling questionnaire. To ensure quality of the responses, care was taken by Censuswide to remove panel members suspected of being career respondents, completing surveys with speed, or entering contradictory or false data. Panelists understood that they were free to drop out at any point in accordance with the MRS rules [19]. Accordingly, no responses from “part completed” interviews were included in the results since it could not be guaranteed that the respondent had not revoked their consent to participate. Respondents had the opportunity to go back to previous items and change their answers.

The panels were recruited with the aim of having a balanced sample across all demographics, and the invitation was sent out randomly to achieve a broad sample. In the panel, the number of participants were proportional to the number of inhabitants of each country region. In the study population, the number of respondents of a given country region was proportional to that region. Thus, there was an attempt to create a representative panel. The panel of each country surveyed was characterized by region of residence, gender, and age, and—for the UK only—with regard to socioeconomic status (SES).

Censuswide continued to recruit respondents until reaching the target of at least 1000 respondents per country; thus, the absolute number of surveys sent per country was based on the country’s response rate.

This survey met the requirements for institutional review board exemption as listed in 45 CFR 46.101(b). Participant information was recorded in such a manner that the human subjects involved cannot be identified.

### Population

The survey population was a sample of people who previously agreed to participate in a social research panel. Invitations to the survey were sent to participants residing in 6 large Western European countries with different reimbursement policies of IVF treatment and different healthcare systems. Demographic information was not a selection criterion, but limited characteristics were recorded. Because the sample was made up of volunteers, females were slightly more represented in the panels than males in most countries, apart from Italy and Germany. In addition, the population aged 16–44 was over-represented in all panels (roughly 70%) compared to the country population (between 45% and 50%). In the UK, the only country where SES was known, the panel was representative of SES of the general population.

### Questionnaire (see S1 Appendix)

The current analysis focuses on IVF reporting on responses to 12 distinct items within the questionnaire that explored respondent opinions on IVF treatment and gamete donation. Specifically, topic areas were acceptance of IVF (including experience using IVF and willingness to use IVF) (Item 1); perceived success rate of IVF (Item 2); age limits regarding use of IVF (Item 3); payment for IVF (public/government funding) (Item 4); number of IVF cycles to be funded (Item 5); criteria that should be used to support government/public funding for IVF (Item 6); payment for IVF (individual couple) (Item 7); opinions related to the use of IVF by individuals who were single (Item 8) or same sex female couples (Item 9); opinions of IVF use likely increasing over the next few years (Item 10); opinions on the potential for delay of conception due to availability of IVF (Item 11); and support for gamete donation (egg and sperm) (Item 12). The remaining 10 questionnaire items relate primarily to fertility preservation and will be reported separately.

### Statistical analysis

Questionnaire responses were analyzed according to sex, age, sexual orientation, and country using SAS version 9.4 (SAS Institute Inc., Cary, NC). Frequencies across responses to individual items and demographic groups using Pearson’s Chi square tests were performed to investigate differences in frequencies between demographic categories (i.e., age group, sex, sexuality, country) and by survey item.

In addition to individual items (See S1 Appendix for questionnaire items and response scales), composite scores were computed for 3 categories. For the 3 categories, attitudes to IVF were measured using items that were combined to form a total favorability score. Items were summed and dichotomized into 0 (no favorable item) to 1 (favorable on at least one item) then assigned to equal binary categories of “low” and “high”. Average numerical values were calculated based on the mean of the midpoint of each value or age band selected by each respondent.

The following 3 categories were analyzed:

1. Beliefs and attitude toward IVF treatment: Item 1 (“Would you/have you ever considered using IVF treatment?”), Item 2 (“What do you think the success rate of becoming pregnant through IVF is?”), and Item 11 (“Do you think that the availability of IVF treatment encourages people to delay conception?”)
2. Funding: Item 4 (circumstances under which IVF treatment should privately or publicly funded), Item 5 (maximum number of rounds of IVF that the government should fund for any individual/couple), and Item 7 (“How much would you pay to have a child through IVF?”)
3. Perceived limit to availability: Item 3 (“What do you think should be the minimum and maximum age limit for acceptance for IVF treatment?”), Item 8 (“Do you believe that IVF treatment should be available to single women without a partner?” [in this situation, sperm would be provided by a donor]), Item 9 (“Do you believe that IVF treatment should be available to same sex female couples?” [in this situation, sperm would be provided by a donor]), and Item 12 (“Do you support the practice of egg donation and sperm donation?”)

Chi square tests were performed to test for differences across each demographic category for each binary concept. Differences were considered significant if the probability of the calculated difference in frequency was less than a value of 0.05, representing a rejection of the null hypothesis of no difference in frequencies between groups.

## Results

### Response rate and demographic characteristics of the sample

A total of 8,682 individuals were contacted via e-mail and asked to participate and 6,610 individuals responded to all questionnaire items (70% response rate). Response rates were highest in the UK and Italy (89% and 90%, respectively) as compared with Sweden and France (55% and 56%, respectively).

Among respondents, there were marginally more males than females (Table 1), and the large majority (88%) was heterosexual. The age groups most represented were 25–34 years and 35–44 years.

**Table 1:** Characteristics of study population.

	UK N (%)	France N (%)	Germany N (%)	Italy N (%)	Spain N (%)	Sweden N (%)	Total N (%)
Total*	1,043 (17)	1,022 (17)	1,011 (17)	1,024 (17)	1,003 (16)	1,005 (16)	6,110 (100)
**Sex**							
Male	408 (39)	440 (43)	562 (56)	454 (44)	611 (61)	674 (67)	3,149 (52)
Female	635 (61)	552 (54)	449 (44)	570 (56)	392 (39)	331 (33)	2,961 (48)
**Age**							
16–24	131 (13)	86 (8)	123 (12)	69 (7)	87 (9)	236 (23)	733 (12)
25–34	298 (29)	198 (19)	247 (24)	246 (24)	285 (28)	329 (33)	1,603 (26)
35–44	205 (20)	293 (29)	224 (22)	318 (31)	338 (34)	221 (22)	1,599 (26)
45–54	205 (20)	242 (24)	209 (21)	245 (24)	199 (20)	125 (12)	1,225 (20)
55+	204 (20)	203 (20)	208 (21)	146 (14)	94 (9)	94 (9)	950 (16)
**Sexual orientation**							
Heterosexual	936 (90)	928 (91)	853 (84)	958 (94)	930 (93)	791 (79)	5,398 (88)
Bisexual	40 (4)	42 (4)	42 (4)	23 (2)	30 (3)	80 (8)	257 (4)
Homosexual	31 (3)	24 (2)	43 (4)	13 (1)	26 (3)	36 (4)	173 (3)
Information not provided	36 (3)	28 (3)	73 (7)	30 (3)	17 (2)	98 (10)	282 (5)

*The distribution of respondents by country is similar despite differences in response rate by country because surveys continued to be sent until the target number of responses (~1,000) had been received from each country.

### Beliefs and attitude toward IVF treatment

**Fig 1:**
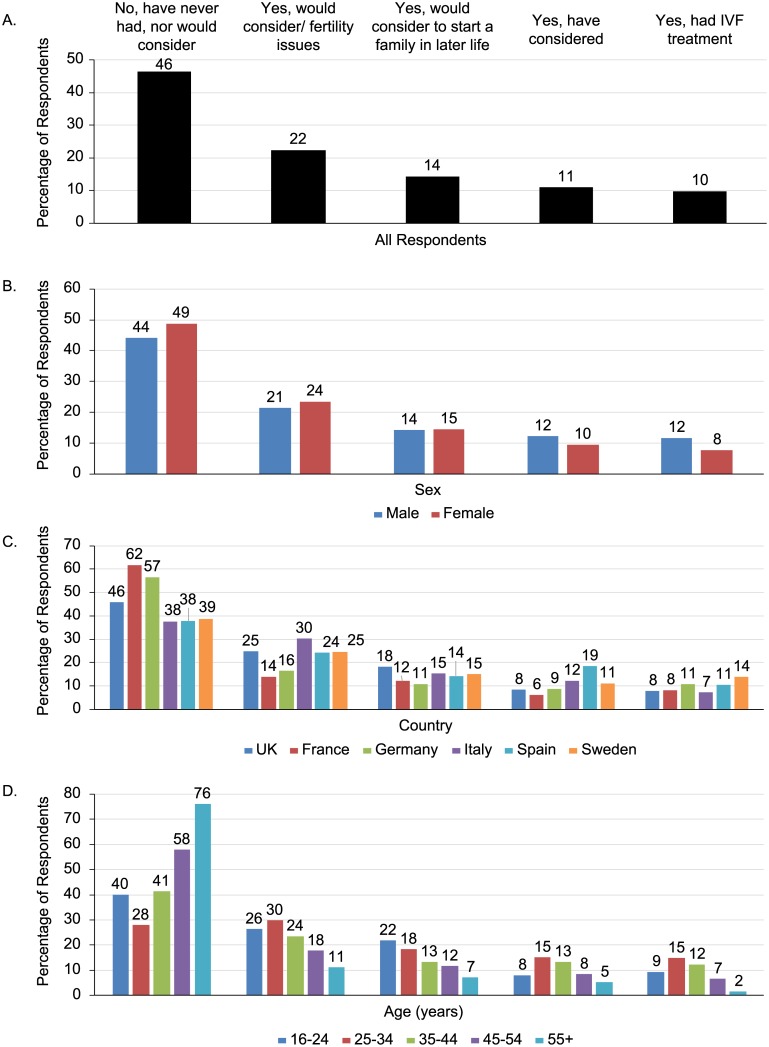
Degree of acceptance of IVF (A) among all respondents; (B) by sex; (C) by country; (D) by age. Respondents could select more than 1 response. Please refer to the questionnaire for full response text.

Regarding acceptance of IVF (Item 1), more than half of the respondents (54%) had considered or would consider having IVF treatments (Fig 1). Nearly 10% of the respondents had undergone IVF treatment. Among the various groups analyzed, a greater proportion of male respondents compared to female respondents (*P*<0.001) and homosexuals (63%) and bisexuals (82%) compared to heterosexual respondents (57%) (*P*<0.003) had IVF or had considered/would consider IVF. Fewer respondents from France and Germany compared with respondents from other countries (*P*<0.001) and a higher proportion of respondents belonging to the youngest age groups (16–24 and 25–34 years) as compared to the older age groups (*P*<0.001) had IVF or had considered/would consider IVF.

When asked to estimate the success rate of becoming pregnant through IVF (Item 2), the overall mean response was 47%. Sixty percent of all respondents estimated IVF success rates as higher than 40%. Only 16% believed that IVF was successful ≤20% of the time. UK respondents gave the lowest mean IVF success rates (40%). Success rates tended to be estimated as higher than 40% more often by men (61%; *P*<0.001), respondents age 25–34 years (63%; *P*<0.001), homosexual respondents (61%, *P*<0.001), and respondents from Spain (79%, *P*<0.001).

In response to Item 11 (“Do you think that the availability of IVF treatment encourages people to delay conception?”), over half of the respondents answered “yes” (52%), with 54% of men (*P*<0.001), 55% of respondents in age groups 25–34 (*P*<0.001), 53% of respondents 55+ years (*P*<0.001), and 59% of respondents from France and Germany (*P*<0.001) responding “yes”. In response to Item 10 (“Do you think the use of IVF treatment will increase over the next 5 years?”), more than 3 respondents out of 4 believe that use of IVF treatment will increase in the next 5 years.

When items were grouped into the category “Attitude and beliefs,” scores from men (*P*<0.001), respondents age 25–34 (*P*<0.001), homosexual respondents (*P*<0.001), and respondents from France and Germany were ranked higher compared with scores from other groups. The lowest ranked attitudes and beliefs were recorded from respondents from Sweden (*P*<0.001).

### Attitudes toward funding

**Fig 2:**
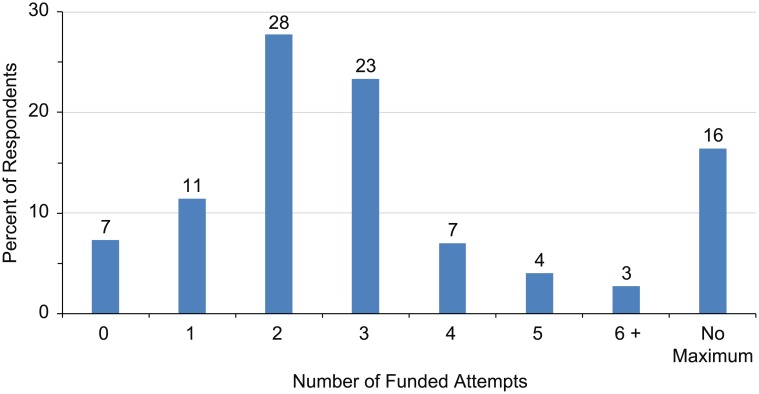
Opinions regarding the number of publicly funded IVF cycles.

Regarding the number of IVF attempts to be publicly funded (Item 5), 93% of respondents answered that at least one IVF cycle should be publicly funded (Fig 2). Overall, the responses indicated that a mean of 2.4 cycles should be publicly funded. By group, the number was higher when answered by women, respondents age 25–34, homosexual and bisexual respondents, and respondents from Sweden and France; all *P*<0.001.

When asked why they believed there should be a maximum limit to the number of publicly funded IVF cycles (Item 6), about half (52%) answered “because there are other ways to have a child” and “Because there are better ways of allocating healthcare funds raised by taxpayer money (i.e., into other medical issues)” (48%).

**Fig 3:**
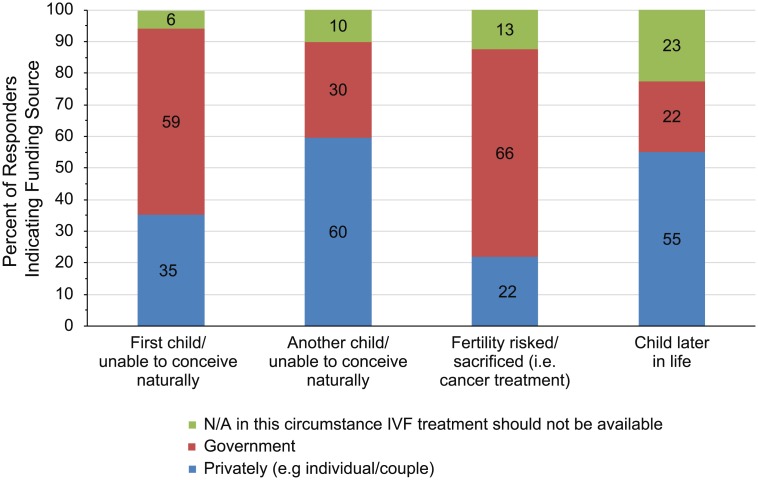
Opinions regarding the funding source for IVF. See questionnaire for full response text.

When asked about circumstances that might influence whether IVF treatment should be privately or publicly funded (Item 4), respondents agreed that funding of treatment should vary with certain circumstances (Fig 3). The majority of respondents agreed that circumstances such as primary infertility and decreased fertility secondary to treatment for medical conditions (i.e., cancer) should be publicly funded. By contrast, after having a first child or when a couple decides to have a child later in life, the majority of respondents indicated that IVF should be financed privately. Of interest, 23% of respondents believed that IVF treatment should not be available to delay parenthood.

**Fig 4:**
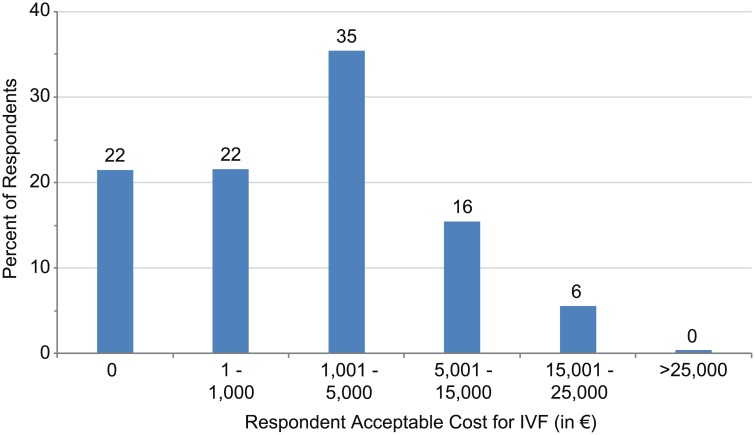
Acceptable cost for IVF (in €). Mean acceptable cost: 5,400 €.

Information about willingness to pay to have a child utilizing IVF (and ranges for amounts they are willing to pay [in €]) (Item 7) is shown in Fig 4. Overall, 57% were willing to pay at least 1,000 €, with a mean amount of 5,400 € for a child brought to life through IVF. More than 20% of all respondents indicated they were unwilling to pay any sum of money for IVF treatment. An even higher proportion of respondents in the age 45–54 (27%) and 55+ (40%) groups and respondents from France (29%) were unwilling to pay for IVF. Respondents willing to pay the highest amount were in the age 16–24 group (mean of 6,800 €), compared to other age groups; the mean amount that respondents from Spain, Sweden, and the UK would pay was similar (6,100 €-6,900 €) and higher than France and Germany (3,600 €-5,000 €). However, when willingness to pay a “high value” was analyzed, no differences were observed in responses according to sex (*P* = 0.056) or sexual orientation (*P* = 0.16).

The analysis of the funding category showed that overall attitude and beliefs toward funding were ranked higher in men, the 2 lowest age groups (16–24 and 25–34), heterosexual respondents, and respondents from Germany, Spain, and the UK (lowest in respondents from Sweden) compared with other groups (*P*<0.001 for all comparisons).

### Perceived limits to availability of IVF treatment and gamete donation

**Fig 5:**
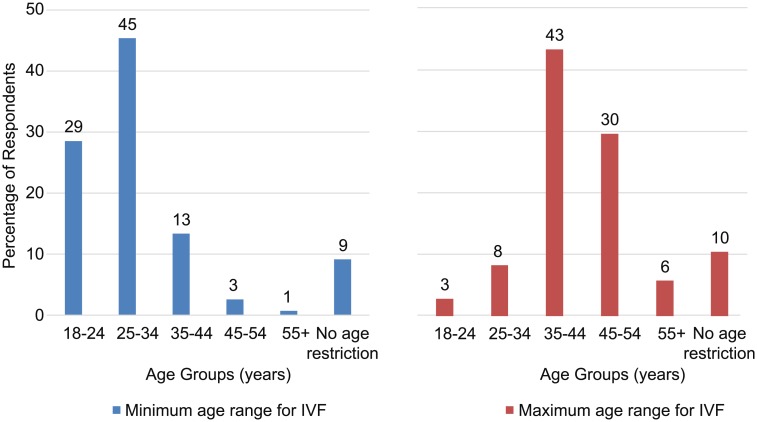
Minimum and maximum acceptable age ranges for IVF.

Respondents’ opinions about the minimum and maximum age for IVF treatment (Item 3) are presented in Fig 5. The mean minimum age for IVF overall was 29 years, while the mean maximum age was 42.

The mean minimum age for IVF was similar between men and women (29 years), was lower in the youngest age group (28 years) as compared to the other age groups (29–30 years) and respondents from Spain (28 years) and was higher in respondents from Sweden (31 years). As for mean maximum age, it was slightly higher in women than in men (43 versus 42 years), in age groups 35–44 and 45–55 (43 years) as compared to other age groups, and in respondents from Spain and Italy (44 years).

Regarding items about whether IVF access should be granted to single women (Item 8) and to same-sex female couples (Item 9), the overall majority agreed that it should (61% and 64%, respectively). More women than men agreed (64% vs 58% about single women and 67% vs 62% about same-sex female couples; *P*<0.001). Respondents from the youngest age group agreed more frequently than those in the oldest age group (70% and 79% in 16–24 age group versus 45% and 47% in 55+ age group, *P*<0.001). Most bisexuals and homosexuals agreed that single women and same sex female couples should have access to IVF, respectively (74%, 71%, and 85%, 81% compared to heterosexuals who agreed 60% and 63% of the time; *P*<0.001). Respondents from Spain had the highest level of agreement with availability of IVF to single women and same-sex female couples (81% and 82%), whereas respondents from Italy had the least (50% and 46%); all *P*<0.001.

A large majority of respondents supported both egg donation and sperm donation (78% for both) (Item 12), by selecting “yes” as their response, with 79% of women and 76% of men supporting egg donation (*P =* 0.02) and sperm donation (*P =* 0.002). More respondents from the two youngest age groups supported egg donation and sperm donation (80% to 81% versus 72% to 77% in the older age groups*; P*<0.001 for both). More bisexuals and homosexuals than heterosexuals supported egg and sperm donations (80% to 88%, *P*<0.002 versus 78% for both). Among the various countries, respondents from Spain supported gamete donation most often and those from Italy least often (92% to 93% versus 61% to 63%; *P*<0.001 for both).

Perceived limits to IVF availability as derived by combining items on age limit, availability of IVF to single women and same sex female couples, and support of gamete donation were ranked higher in women than in men, higher in the two youngest age groups (16–24 and 25–35) as compared to other age groups, higher in homosexuals and bisexuals compared to heterosexuals and higher in respondents from Germany compared to other countries, with the lowest rank recorded from respondents from France (*P*<0.001 for all comparisons).

## Discussion

To our knowledge, the current LIFE survey represents the largest study performed so far concerning public opinion regarding ART with over > 6,000 respondents across Europe. The respondents expressed favorable attitudes in relation to IVF and its success, the need for public funding, the use of IVF among modern families with different lifestyles, and support for gamete donation. The youngest age groups, homosexuals, bisexuals, German respondents, and men had similar overall positive attitudes and beliefs toward IVF and opinions in relation to public funding and perceived limits to availability. Perceived limits to availability were stronger in women. These differences in acceptability, beliefs, and perception should be taken into consideration for future research or when designing awareness campaigns or other initiatives in these groups. The survey revealed over-optimism about success of IVF which may lead to too much reliance on IVF as a mean to overcome infertility.

Previously, fertility knowledge and treatment attitudes were investigated in 10,045 individuals actively trying to conceive (International Fertility Decision-Making Survey, IFDMS) [21]. Both positive (safety, efficacy) and negative views (physical/emotional effects) on fertility treatments were reported. Negative beliefs were strongly associated with higher fertility knowledge and were mostly observed in women, people with a university education, with employment, living in the more economically developed countries, childless individuals and those trying to conceive for more than a year. Although the items used to assess attitude toward IVF treatment were different in our survey, current results clearly indicate a more favorable attitude. One explanation may be the different surveyed populations for LIFE and the IFDMS. The IFDMS largely represented people with [failed] fertility treatment experience (about 70%), whereas the LIFE survey comprised an untreated sample from the general population with little treatment experience (<10%). It is also possible that social normative view has changed over time as IVF has become more main stream technology.

Variations in attitudes towards IVF were observed in our survey but these appeared motivated by specific interests of certain groups, such as ones more interested in fertility (i.e., younger respondents and females). Willingness to pay for IVF was highest in the age groups that are likely to be most concerned about parenthood, with respondents younger than age 35 willing to pay the highest amount, and 32% of respondents age 45 or older unwilling to pay any amount for IVF. A previous survey found men less supportive of IVF than women and reported that when respondents knew someone with infertility problems, they were more likely to support reimbursement [22]. Interestingly in our survey more men (23.9%) than women (17.2%) reported that they or their partner had considered or had IVF.

The majority of respondents of our survey agreed that IVF should be publicly funded to some extent, with the strongest support (66%) for public funding for infertility due to a disease or its treatment (i.e., cancer treatment) or for primary infertility. In general, support for public funding was consistent with results reported previously in a survey of European respondents in which the cost of IVF was presented as being similar to that of a hip replacement [22]. Thus, government reimbursement for IVF may garner additional support if the public is educated to understand that infertility is a medical, rather than social issue. Fifty-nine percent of respondents believed public funding should be limited to one child. This may be linked to the fact that, in most of the countries surveyed, the financial burden of infertility treatments is partly alleviated by the social security system. This is particularly true in France where infertility treatments are fully reimbursed and where willingness to pay (WTP) is the lowest of all countries. WTP in our survey may have been biased due to the low starting point (0 €) that was presented to the respondents. A recent paper that explored the reliability and validity of surveys for estimating willingness to pay [23] found that a strong anchoring/starting point bias was evident in surveys that presented a low starting value, with lower WTP values obtained.

Overall, we observed strong support by respondents of most countries to permit IVF access for single women and same-sex female couples. More respondents in our survey supported egg and sperm donation (78%) than were willing to consider IVF for themselves (46%), suggesting societal support for the procedure, even among respondents that would not undergo it. There were differences in responses depending on the country of residence and this factor may reflect variation in cultural, social, and religious norms [23]. For example, 38% of respondents from Italy (with a large Catholic influence) would not consider IVF and were the least supportive of IVF and of gamete donation.

In Sweden, the law changed in April 2016 to permit IVF access for single and same sex women; access is also permitted in Germany, Spain, and the UK, but not in France and Italy [24]. The results of a study examining the impact of regulations and public financing in Europe on the utilization of IVF showed that countries providing the most generous public financing tend to restrict access [25]. Furthermore, an international survey on the impact of affordability on access to fertility treatment conducted in 2014 analyzed data from 30 high and upper-middle income countries (which included the countries in the present study) and showed that lower costs for infertility treatments were positively correlated with an increase in utilization [26]. Thus, the findings from this current survey, which shows support for reimbursement of IVF, may be useful in convincing legislators that a change in public policy to provide more financial support for IVF may be acceptable to their constituents and that lessening of restrictions to access could increase utilization. This could help alleviate population decline and its associated implications [24].

One issue of concern is over-optimism about IVF success as views could affect childbearing efforts and delay pregnancy to later in life when fecundity is suboptimal. Although the item investigating opinion on success rates in our survey did not specify whether it was related to a single fresh IVF cycle, additional pregnancy chances using cryopreserved embryos, or the use of multiple IVF cycle, respondents’ expectations of IVF success appear to be high as reflected by an estimated success rate of IVF of 47%. Actual pregnancy rates following IVF are reported to be 25% to 35%, per single IVF cycle and fresh embryo transfer [27], suggesting that our respondents, on average, had falsely high expectations about the success rate concerning IVF treatment.

Postponing childbirth has become common in Western countries especially among college-educated women [28]. Over-optimism of IVF success rates may lead to over-reliance on IVF as way of compensating for infertility, especially when due to delayed childbearing [29–31]. More than half of the respondents in this survey believed that the availability of IVF treatment encourages a delay in conception. Education of young men and women about age-related decrease in fertility as well as IVF procedures and success rates over single versus multiple cycles may help inform family planning and reduce reliance on fertility interventions.

Limitations of our survey include the possibility that the population responding may not be representative of the actual population. Indeed, respondents were invited from volunteer participants of an online panel [18]. In general, information on employment, socioeconomic, and professional status of the respondents is lacking. Data on fertility status were not collected, except to determine that 20% of the respondents had either considered or undergone IVF treatment and are likely to have had (or at least know someone with) fertility issues. Respondents with IVF experience may be less supportive if their experience was with failed IVF [23]. Given that we disproportionately sampled from people in childbearing age, there potentially is an over-representation of people interested in fertility. A recent survey examined the attitudes of 146 fertile vs 93 infertile women in Lithuania and found that fertile respondents were more likely to believe that IVF should be limited to married couples within a defined age limit and should include a psychological assessment [32]. Infertile couples were more likely to view public funding as a key component of IVF treatment. Thus, it is unknown to what extent key factors such as fertility status would have affected the responses in the current survey. The attitudes of people older than age 55 are of questionable value for several of the survey items; however, the majority of the survey respondents were of child-bearing age. Although willingness to pay for IVF may not be as relevant for the youngest or oldest respondents, inclusion of the entire population is helpful in gauging support for public funding. Our analysis was designed as univariate with the intention to keep it simple. The purpose of the survey is to provide the general European view from the largest countries in Western Europe, and it was not planned to explore or highlight differences between countries. Thus, each country’s data were not weighted by country population size; however, Sweden is the only country with a markedly different population size. Due to the number of countries, demographic variables, and relatively large number of questions, our objective is to provide a straightforward reporting of the results of this large survey. The lack of information on social beliefs and socioeconomic characteristics did not allow us to determine the impact of social variables on our findings and also limited our ability to conduct further analyses that may have explored reasons for restriction of IVF access for younger age groups or to one pregnancy. Finally, the average time it took respondents to complete the survey (7.5 min) was short and this factor likely contributed to the high response rate (6,110/8,682, 70% response).

The LIFE survey is one of the largest to date and provides information about the public’s perception on IVF treatment, its acceptance, funding, and limits to availability. Our multinational sample covered countries where policies regarding treatment reimbursement are different. The survey results demonstrate an overall positive attitude of the general population toward IVF and toward public funding of infertility and fertility treatment among European respondents. These findings may potentially be utilized to guide and inform discussions among patients and prescribers and among legislators and payers for the funding of these procedures.

## Supporting information

S1 Appendix Study questionnaire. (DOCX)
