# Supplementary material for: Beliefs, attitudes and funding of assisted reproductive technology: Public perception of over 6,000 respondents from 6 European countries
Source: PLoS One. 2019 Jan 25;14(1):e0211150. doi: 10.1371/journal.pone.0211150 (PMC6347360; doi:10.1371/journal.pone.0211150)
Supplement: S1 Appendix — (DOCX) [file pone.0211150.s001.docx]

| **In vitro fertilisation (IVF) is a process used by individuals or couples who are unable to conceive naturally. The process involves a small amount of a woman’s eggs being removed from her ovaries and fertilised with sperm in a laboratory. If an egg is successfully fertilised it will be implanted into the woman in the hope that she will become pregnant.** |
| --- |
| **Item 1.** **Would you/have you ever considered using IVF treatment?**  Tick all that apply   - Yes, I/my partner have/has had IVF treatment - Yes, I/my partner have/has considered IVF treatment (but did not do it) - Yes, I would definitely consider IVF treatment in the future, if I/my partner had fertility issues - Yes, I (or wish that my partner?) would consider IVF treatment in the future to start a family in later life (but are not facing fertility issues now) e.g. social freezing - No, I have never had, nor would I ever consider IVF treatment |
| **Item 2. What do you think the success rate of becoming pregnant through IVF is?**  Possible answers given:   - 0-10% - 11-20% - 21-30% - 31-40% - 41-50% - 51-60% - 61-70% - 71-80% - 81-90% - 91-100% |
| **Item 3. What do you think should be the minimum and maximum women age limit for acceptance for IVF treatment?**   - Minimum - 18-24 - 25-34 - 35-44 - 45-54 - 55+ - N/A I do not think there should be restrictions based on age - Maximum - 18-24 - 25-34 - 35-44 - 45-54 - 55+ - N/A I do not think there should be restrictions based on age |
| **Item 4. In what circumstances do you think that IVF treatment should be funded privately (e.g. by individual/couple) or by the government?**   - When having a first child and unable to conceive naturally - Privately (e.g. individual/couple) - Government - N/A in this circumstance IVF treatment should not be available - When having another child (in addition to any they currently have) and unable to conceive naturally - Privately (e.g. individual/couple) - Government - N/A in this circumstance IVF treatment should not be available - When fertility has been risked/sacrificed (e.g. due to cancer treatment) - Privately (e.g. individual/couple) - Government - N/A in this circumstance IVF treatment should not be available - When the decision has been made to have a child later in life (i.e. when fertility decreases) - Privately (e.g. individual/couple) - Government - N/A in this circumstance IVF treatment should not be available |
| **Item 5. What do you think should be the maximum number of rounds of IVF that the government should fund for any individual/couple?**   - 0 - 1 - 2 - 3 - 4 - 5 - 6 + - There should not be a maximum limit |
| **Item 6. Why do you believe there should be a maximum limit?**  Tick all that apply   - Because there are other ways to have a child (i.e. adoption/surrogacy) - Because there are better ways of allocating healthcare funds raised by taxpayers money (i.e. into other medical issues) - Because of the potential psychological impact on the individual/couple - Other, please specify |
| **Item 7. How much would you pay to have a child through IVF?**   - €0 (£0) - €1 - €1,000 (approx. £0.86p - £855) - €1,001 - €5,000 (approx. £856 - £4,276) - €5,001 - €15,000 (approx. £4,277 - £12,828) - €15,001 - €25,000 (approx. £12,829 – £21,381) - €25,001+ (approx. £21,382+), please specify in £ €25,001+ (approx. £21,382+), please specify in £ |
| **Item 8. Do you believe that IVF treatment should be available to single women without a partner (in this situation, sperm would be provided by a donor)?**   - Yes - No |

| **Item 9. Do you believe that IVF treatment should be available to same sex female couples (in this situation, sperm would be provided by a donor)?**   - Yes - No |
| --- |
| **Item 10. Do you think the use of IVF treatment will increase over the next 5 years?**   - Yes, it will increase significantly - Yes, it will increase slightly - No, it will stay the same - No, it will decrease |
| **Item 11. Do you think that the availability of IVF treatment encourages people to delay conception?**   - Yes - No |
| **Item 12. Do you support the practice of egg donation and sperm donation?**   - Egg donation - Yes - No - Sperm donation - Yes - No |
| **Qi. What gender are you?**   - Male - Female |
| **Qii. Are you aged?**   - 16-24 - 25-34 - 35-44 - 45-54 - 55+ |
| **Qv. Which of the following best describes your sexual orientation?**   - Heterosexual - Bisexual - Homosexual - I do not wish to divulge this information |
